# Alpha-synuclein structural variants driving tau pathology and disease interaction

**DOI:** 10.4103/NRR.NRR-D-25-01192

**Published:** 2026-01-02

**Authors:** Chuanqi Sun

**Affiliations:** Department of Neurology, David Geffen School of Medicine, University of California, Los Angeles (UCLA), Los Angeles, CA, USA; Departments of Biological Chemistry and Chemistry and Biochemistry, UCLA-DOE Institute, UCLA, CA, USA

**Redefining neurodegenerative diseases: from isolated protein pathologies to intertwined disease networks:** A common feature of neurodegenerative diseases such as Parkinson’s disease (PD), Alzheimer’s disease (AD), Lewy body dementia, and multiple system atrophy is the accumulation of misfolded, insoluble protein aggregates in specific neurons or glial cells (Buchholz et al., 2025). In AD, these aggregates are mainly manifested as neurofibrillary tangles composed of Tau protein, while in PD, they are mainly manifested as Lewy bodies composed of α-synuclein.

α-Synuclein is a protein composed of 140 amino acids, which is mainly enriched in presynaptic terminals and plays an important role in synaptic vesicle trafficking, SNARE complex formation and neurotransmitter release. Tau protein is a microtubule-associated protein that is mainly present in axons and is essential for microtubule assembly, stabilization, and axonal transport. Under physiological conditions, both proteins are intrinsically disordered proteins in the soluble monomeric state, but they can undergo conformational changes to form oligomers and fibrils rich in β-folds (Jonaitis et al., 2024).

Neurodegenerative diseases are often classified as separate proteinopathies based on their primary pathological hallmarks (e.g., PD is classified as a synucleinopathy and AD is classified as a tauopathy/amyloidopathy). However, early neuropathological observations have begun to challenge this isolated view. For example, α-synuclein-positive inclusions have been found in some tauopathies, and conversely, tau-positive inclusions have been found in synucleinopathies. Specifically, paired helical filaments of tau have been found at the Lewy body border in autopsy brains of PD patients.

The growing evidence of co-localization and coexistence of α-synuclein and tau pathology suggests that there are significant interactions and synergies between these misfolded proteins that may amplify pathogenic mechanisms and exacerbate neuronal dysfunction. As a result, the field is undergoing a paradigm shift from viewing neurodegenerative diseases as isolated proteinopathies to understanding them as breakdowns in proteostasis networks driven by complex molecular interactions. This broader understanding of neurodegenerative diseases, that their core problem is a systemic failure of cellular protein quality control rather than just isolated protein misfolding, fundamentally reorients diagnostic and therapeutic strategies (Katorcha et al., 2017). It requires that diagnostics move toward multi-biomarkers to identify overlapping pathologies, and that therapeutic development focus on integrated strategies that address common pathogenic mechanisms or restore overall protein homeostasis rather than targeting single proteins in isolation. This has profound implications for the design of future clinical trials and personalized medicine.

**Protein “strains” and the concept of cross-seeding:** Both α-synuclein and Tau proteins exhibit “prion-like” properties. This means that they can misfold, aggregate, and then act as “seeds” to induce templated misfolding and aggregation of their soluble native monomers, leading to the propagation of pathology. Traditionally, “prion-like” propagation has focused on the self-templated mechanism of misfolded proteins inducing misfolding of their own soluble counterparts. However, the study clearly points to the concept of “cross-seeding,” that is, α-synuclein aggregates can induce Tau protein aggregation. This shows that the template mechanism is not strictly limited to homologous proteins, but can occur between different amyloid proteins, suggesting the existence of shared structural features or interaction motifs between different amyloid fibrils. This broader understanding of prion-like behavior suggests that there is a more general underlying pathological process in neurodegenerative diseases, in which one misfolded protein can initiate or accelerate the pathology of another protein, leading to a complex, multi-protein aggregation cascade (Guo et al., 2013).

Crucially, α-synuclein can exist as different “strains” that are conformational variants of its fibrils with distinct biochemical properties and biological activities, including differences in cross-seeding efficiency toward Tau aggregation. This conformational diversity is thought to be a key factor contributing to the tremendous clinical and pathological heterogeneity observed in synucleinopathies, including PD, Lewy body dementia, and multiple system atrophy. Cross-seeding specifically refers to the phenomenon whereby aggregates of one amyloid protein, such as α-synuclein, are able to induce misfolding and aggregation of an unrelated protein, such as Tau, but not its own soluble counterpart. The central scientific question is to elucidate the precise structural basis and complex molecular mechanisms by which specific α-synuclein strains directly promote Tau aggregation, leading to the complex and overlapping pathologies observed in neurodegenerative diseases such as AD, PD, and Lewy body dementia. Understanding this cross-seeding is critical for developing integrated and effective therapeutic strategies.

**Structural insights into α-synuclein strain B and its interaction with tau pathology: a molecular bridge:** Cryo-electron microscopy has revolutionized the field by providing atomic-resolution structural information of amyloid fibrils, including α-synuclein (Sun et al., 2025). A recent landmark study (“Structural basis of a distinct α-synuclein strain that promotes tau inclusion in neurons”) identified and characterized a novel α-synuclein fibril polymorph, named “strain B.”

The structure of strain B was solved at 2.6 Å resolution by helical reconstruction cryo-electron microscopy. A notable feature of strain B is its unique fibril core, which prominently integrates both the N- and C-termini of α-synuclein, which is different from previously characterized α-synuclein fibril structures. Most previously characterized α-synuclein fibril structures primarily involve its central non-amyloid component region. Strain B uniquely integrates both the N- and C-termini into its fibril core, representing a significant structural deviation. This unique conformation, and in particular the key role of the C-terminal segment (D105-E115) in its formation and interaction with Tau, suggests a unique binding interface. Therefore, inhibitors designed specifically to target this N/C-terminus-encompassing core or D105-E115 region could provide a highly selective approach to mitigate Tau co-aggregation driven by this particular α-synuclein strain, potentially minimizing off-target effects compared to more general anti-aggregation strategies. This insight has driven the development of precision medicine based on structural polymorphisms.

The discovery of α-synuclein strain B is its ability to directly promote tau aggregation. Tau deposition is a hallmark of AD and other tauopathies. This direct interaction provides a key molecular link between synucleinopathies and tauopathies, two major neurodegenerative diseases previously thought to be distinct in their primary protein pathology (Tarutani et al., 2023).

The finding of α-synuclein-positive inclusions in some tauopathies and, *vice versa*, tau-positive inclusions in synucleinopathies has long suggested an overlap or “cross-talk” in the pathologies. Strain B now provides a concrete structural and mechanistic explanation for this phenomenon, suggesting that the co-presence of these misfolded proteins can amplify the pathogenic mechanism.

**Figure 1:**
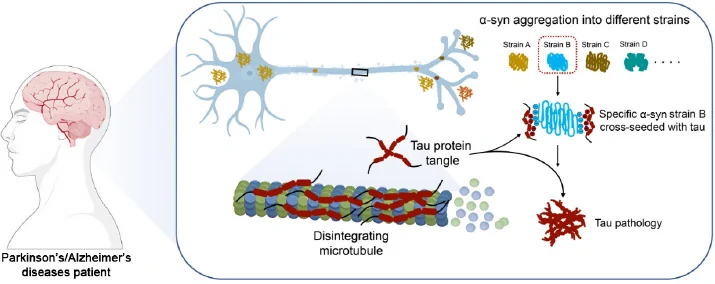
Proposed mechanism of α-synuclein (α-syn) strain-specific cross-seeding of tau in Parkinson’s/Alzheimer’s diseases. This figure illustrates the pathological progression in patients with Parkinson’s and Alzheimer’s diseases, highlighting the role of α-syn aggregation and its interaction with tau protein. Initially, α-syn aggregates into distinct strains (such as Strain A, B, C, D), represented by different colored fibrils. A specific α-syn strain, particularly Strain B (highlighted in red dotted box), is shown to cross-seed with normal tau protein. This interaction leads to the aggregation of tau protein into insoluble tau protein tangles, which are detrimental to neuronal function. The tau tangles then disrupt and lead to the disintegration of microtubules, crucial structures for intracellular transport and neuronal stability. Ultimately, this cascade results in widespread tau pathology, contributing to neurodegeneration observed in Parkinson’s and Alzheimer’s diseases. The neuron illustration depicts the location of these pathological events within the cell. These illustrations were created with biogdp.com based on the original data from Jiang et al. (2025).

A fragment of the C-terminal domain of α-synuclein (D105-E115), identified as key for the formation of strain B, is postulated to play a key role in its interaction with tau. This suggests the existence of a specific molecular interface that mediates cross-seeding or co-aggregation (**Figure 1**).

PD (α-synuclein) and AD (tau) are two distinct neurodegenerative diseases. However, there is a clinical and pathological overlap: α-synuclein inclusions are found in tauopathies and tau inclusions in synucleinopathies. This suggests the existence of “cross-talk,” but its mechanisms were previously poorly understood. New findings now show that strain B directly promotes tau aggregation. Not only does this mean that strain B is a molecular bridge between α-synuclein and tau pathology, but more importantly, it suggests a causal relationship whereby one misfolded protein (α-synuclein strain B) actively drives the misfolding of another (tau). This mechanism could explain why patients often present with mixed pathologies and why disease progression is so rapid in some cases (e.g., multiple system atrophy).

Strain B shifts the view of neurodegenerative diseases from a single protein disease to a complex, interconnected protein pathology. It suggests that specific strains such as strain B may act as “master promoters” or “amplifiers,” accelerating pathology across multiple protein systems. This has profound implications for understanding disease progression, as targeting one protein (e.g., α-synuclein) may inadvertently affect another (e.g., tau) if the specific strain is not considered. It also emphasizes the need for multi-target therapeutic approaches.

**Therapeutic and diagnostic significance: opening future directions:** In-depth analysis of the structural features of α-synuclein strain B, especially its unique N-terminal and C-terminal core conformations and the key role of the D105–E115 fragment, revealed new therapeutic targets. Unlike previous strategies that widely inhibited α-synuclein aggregation, new ideas can focus on specifically blocking the formation of strain B fibrils or interfering with its conformation-specific interaction with tau, thereby improving the precision and efficacy of intervention (Li et al., 2025).

Understanding why α-synuclein misfolds to form specific structures, such as strain B, will help develop new therapies that can slow or prevent disease progression. Currently, many PD drug candidates still generally target α-synuclein aggregation, but do not distinguish between its conformational polymorphisms. Strain-specific intervention strategies, such as designing small molecules or peptide drugs that can bind to the D105–E115 region, can specifically block strain B formation or its template activity on tau. In addition, regulating the receptor-mediated mechanism that mediates the cross-cellular spread of pathological α-synuclein species also provides an important entry point for future treatment.

More and more studies have shown that different α-synuclein strains may correspond to different clinical phenotypes and disease progression pathways, so they have the potential to become disease biomarkers (Yarbro et al., 2025). Detecting and quantifying specific conformations such as strain B in cerebrospinal fluid or autopsy tissue may help to diagnose synuclein diseases early and accurately, distinguish different subtypes, and even predict disease development trends or treatment responses. This is consistent with the overall goal of developing truly disease-modifying Parkinson’s disease therapies.

The direct structural connection between strain B and tau aggregation not only deepens our understanding of the cross-mechanism between PD and AD, but also shows that these neurodegenerative diseases are not independent of each other but are highly intertwined protein pathology networks at the molecular level. We need to shift from the traditional “single protein pathology” model to a more comprehensive “conformational strain-driven” perspective to identify and target the key drivers behind conformational diversity. This idea can not only explain the phenotypic diversity of the disease but also provide a theoretical basis for precise intervention.

Currently, most treatments for neurodegenerative diseases focus on relieving symptoms, and strategies that can truly delay or reverse the course of the disease are still extremely limited. In the past, the general targeting of α-synuclein aggregation may not cover all pathogenic strains and may even have little effect in pathologies dominated by certain strains. Therefore, a more promising direction in the future is to build a precision treatment system based on the “specific α-synuclein conformation strains existing in the patient’s body.” For example, the development of specific drugs targeting the unique C-terminus of strain B and the tau binding interface is expected to achieve better efficacy in patients with such pathological characteristics. This also suggests that we urgently need to develop diagnostic tools for identifying the presence of specific α-synuclein strains in the body as a prerequisite for individualized treatment.

The establishment of this strain-specific treatment and diagnostic strategy will promote the field of neurodegenerative diseases from the traditional “symptom relief” stage to a new era of “pathological typing-precision intervention.”

**Prospective — A paradigm shift in neurodegenerative disease research:** Neurodegenerative disease research is undergoing a profound paradigm shift, from the traditional concept of protein aggregation as a unified pathological endpoint to focusing on the conformational diversity of misfolded proteins and their “strain”-specific pathological mechanisms. Taking α-synuclein strain B as an example, its unique N-terminal and C-terminal core conformations and its ability to directly promote tau aggregation not only reveal its highly specific pathogenic potential, but also provide strong structural evidence for the prion-like strain hypothesis. This finding shows that α-synuclein strains with different conformations can drive different clinical phenotypes, disease progression rates and even co-pathological processes. Misfolded proteins are no longer the end products of structural chaos, but pathogenic conformational units with intrinsic order and functionality. The specific conformation determines its performance in cellular uptake, transcellular propagation, aggregation dynamics, and interaction with other proteins (such as tau), thereby shaping the phenotype and development trajectory of the disease.

This emerging concept provides theoretical support and a practical path for precision medicine for the diagnosis and treatment of neurodegenerative diseases. In the future, identifying and characterizing the types of α-synuclein conformational strains in individual patients will become the key to disease typing, early diagnosis, patient stratification, and efficacy prediction. In particular, the development of sensitive diagnostic tools that can detect specific pathogenic strains under *in vivo* conditions will significantly improve clinical operability. At the therapeutic level, small molecules, antibodies, or peptide drugs designed around specific strains can achieve precise targeted intervention of pathogenic conformations, thereby avoiding nonspecific side effects and efficacy differences that may be caused by “pan-inhibition” strategies (Mak et al., 2025). The structural “language” of different α-synuclein strains is becoming a key to interpreting the molecular mechanisms and clinical heterogeneity of neurodegenerative diseases. By identifying and translating these “structural dialects,” we hope to advance the treatment model of neurodegenerative diseases from traditional symptomatic support to personalized intervention based on pathological mechanisms and achieve true disease modification or even reversal.
